# Differentiating nonradiographic axial spondyloarthritis from its mimics: a narrative review

**DOI:** 10.1186/s12891-022-05073-7

**Published:** 2022-03-12

**Authors:** Philip Mease, Atul Deodhar

**Affiliations:** 1 Swedish Medical Center/Providence St. Joseph Health and University of Washington, Seattle Rheumatology Associates, WA Seattle, USA; 2 Division of Arthritis and Rheumatic Diseases, Oregon Health & Science University, Portland, OR USA

**Keywords:** Differential diagnosis, Management, Narrative review, Nonradiographic axial spondyloarthritis, Treatment

## Abstract

**Background:**

Optimal treatment of nonradiographic axial spondyloarthritis depends on accurate and timely diagnosis of the underlying disease; however, patients present with common symptoms that, in the absence of radiographic changes, may confound diagnosis.

**Methods and findings:**

In this narrative review, a PubMed literature search was conducted through January 2021, with no date limits, to identify English-language publications discussing classification of nonradiographic axial spondyloarthritis, with an emphasis on clinical features and presentation, differential diagnoses, and mimics of disease. This review describes the epidemiology, clinical features, and burden of disease of nonradiographic axial spondyloarthritis as it relates to the overall axial spondyloarthritis spectrum and discusses mimics and differential diagnoses of nonradiographic axial spondyloarthritis that should be considered when evaluating patients with suspected nonradiographic axial spondyloarthritis in clinical practice.

**Conclusions:**

Recognition of clinical features of nonradiographic axial spondyloarthritis, along with an understanding of comorbid conditions such as fibromyalgia, allows for differentiation from its mimics. Appropriate diagnosis of nonradiographic axial spondyloarthritis is important for aggressive management of disease to reduce pain, avoid loss of function, and improve quality of life.

## Background

Axial spondyloarthritis (axSpA) is an inflammatory disease continuum that ranges from nonradiographic axSpA (nr-axSpA) to radiographic axSpA (r-axSpA), also known as ankylosing spondylitis (AS) [1]. The condition is defined by axial joint involvement, often sacroiliitis, but peripheral arthritis and extra-articular involvement (uveitis, inflammatory bowel disease [IBD], enthesitis, and psoriasis), which are shared with other types of spondyloarthritis (SpA), are quite common [2]. Patients with nr-axSpA vs AS are distinguished by the absence vs presence of definitive sacroiliitis on plain radiographs [3]. Estimates of the prevalence of axSpA and its subtypes are variable. In the United States, the 2009–2010 National Health and Nutrition Examination Survey estimated the prevalence of axSpA in the adult population at 0.9 to 1.4%, with the prevalence of r-axSpA and nr-axSpA estimated to be similar at approximately 0.5% each [4]. Unlike AS, which is male predominant, the prevalence of nr-axSpA is similar or even slightly higher in women than in men [5]. Patients present with symptoms common to other conditions, which can confound diagnosis, particularly in the absence of radiographic sacroiliitis (as in nr-axSpA). As a result, due to delays in timely referral to rheumatology, axSpA has one of the longest diagnostic delays in rheumatology (mean, 6.7 years), with more recent evidence showing that the delay to diagnosis in some countries may be decreasing over time [6]. Timely diagnosis of nr-axSpA is further complicated by lack of radiographic changes, which may never develop, and optimal treatment outcomes are dependent on accurate and timely diagnosis of the underlying disease.

Here, we provide an overview of the current understanding of nr-axSpA, with a focus on its proper diagnosis and distinguishing clinical features in light of the variety of potential presenting symptoms and mimics. This narrative review was developed for rheumatologists and the wider population of healthcare providers (eg, primary care providers, physiatrists, orthopedists, pain specialists, physical therapists, neurologists/neurosurgeons, general physicians, and associated nurse practitioners and physician assistants) who should be aware of nr-axSpA, its mimics, and the potential need to refer to a rheumatologist. We begin with a brief overview of axSpA and the classification of nr-axSpA within this disease spectrum. Subsequently, we discuss the clinical diagnosis of nr-axSpA with a focus on its mimics and differential diagnoses. Finally, we briefly outline the management and treatment of nr-axSpA.

## Main text

### Search strategy

A series of PubMed searches between August 2020 and January 2021 were conducted to identify English-language publications of interest. Search terms included (nonradiographic axial spondyloarthritis OR nr-axSpA) AND (classif* OR delay OR mimic* OR differential diag*), with no date limits. Publications mentioning classification of nr-axSpA, clinical features and presentation (including differences between men and women and between nr-axSpA and r-axSpA), differential diagnoses, and nr-axSpA mimics were considered for inclusion. Additional searches were conducted to further probe specific differential diagnoses. Articles deemed irrelevant based on study type or content diverging from topics of interest were excluded from consideration through review of the title and abstract. Publications cited within relevant articles, as well as any additional studies identified by the authors, were included based on these criteria.

### The spectrum of SpA diseases

Nr-axSpA is a part of the spectrum of axSpA [1], which itself belongs to the wider group of SpA (including psoriatic arthritis, IBD-associated arthritis, peripheral SpA, reactive arthritis, and undifferentiated SpA), which are genetically linked to each other [7] and other immune-mediated inflammatory diseases (including psoriasis and IBD [ulcerative colitis and Crohn’s disease]) [8, 9]. The lifetime probability of progression from nr-axSpA to AS has been estimated to be 50% [10]. An estimated 5 to 10% of patients with nr-axSpA will develop structural changes in the sacroiliac joints indicative of AS over 2 years, increasing to 5 to 40% within 10 years of disease onset [10–12].

**Fig. 1:**
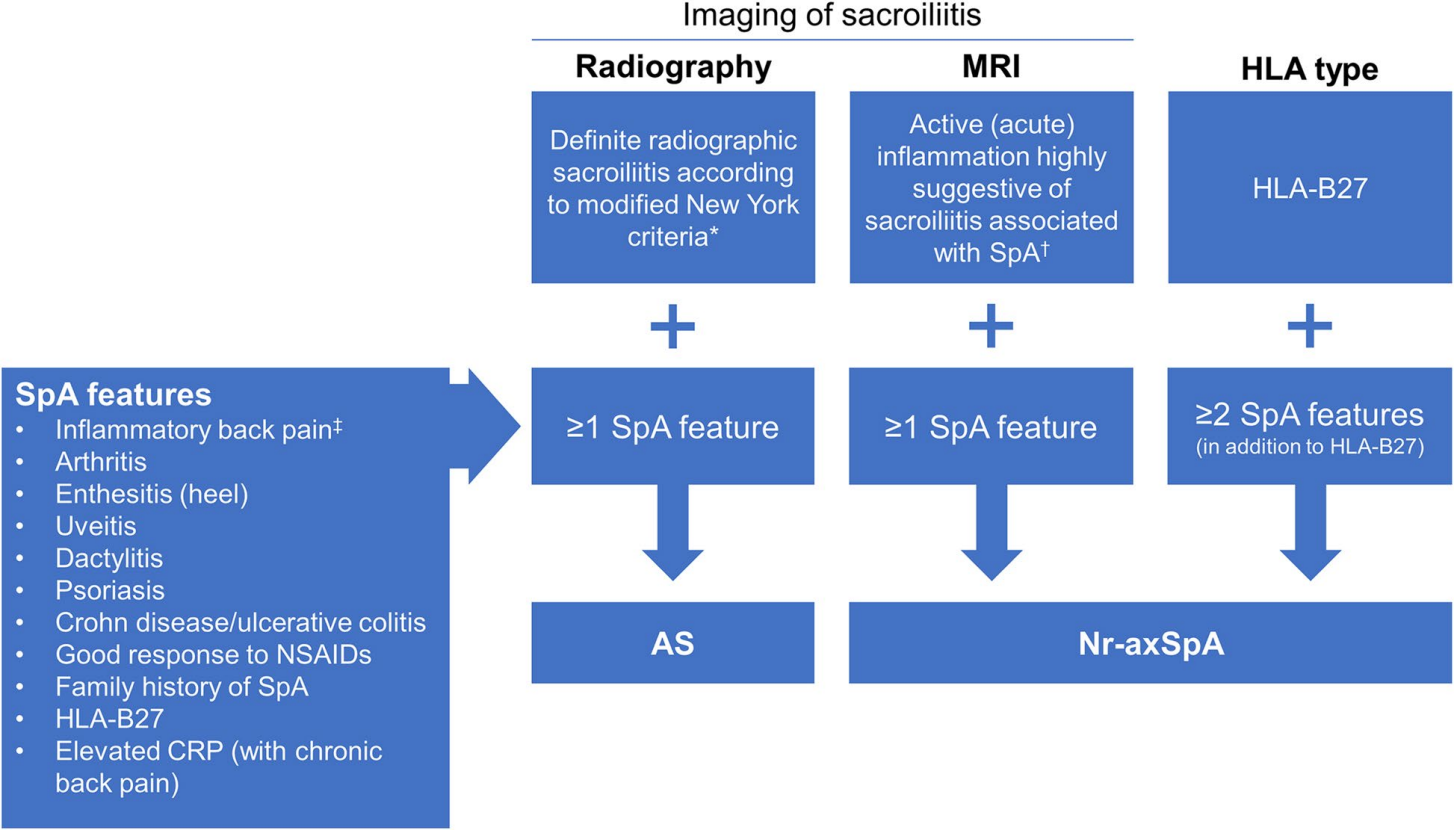
Classification of nr-axSpA and AS using the ASAS criteria for axSpA in patients with back pain lasting ≥3 months and age at onset < 45 years [14]. AS = ankylosing spondylitis; ASAS = Assessment of SpondyloArthritis international Society; CRP = C-reactive protein; HLA = human leukocyte antigen; MRI = magnetic resonance imaging; nr-axSpA = nonradiographic axial spondyloarthritis; NSAID = nonsteroidal anti-inflammatory drug; SpA = spondyloarthritis. * The modified New York criteria for sacroiliitis are: grade ≥ 2 bilaterally or grade ≥ 3–4 unilaterally [13]. .^†^ Active acute inflammation is defined by clearly present bone marrow edema (on short-T1 inversion recovery) or osteitis (on T1 post gadolinium) highly suggestive of SpA, located in subchondral or periarticular bone marrow [15]. ^‡^ ASAS criteria for inflammatory back pain criteria include 4 of the 5 following: improvement with exercise, pain at night, insidious onset, age at onset < 40 years, and no improvement with rest [16, 17]

Since 1984, AS has been classified using the modified New York criteria [13], which require radiographically definitive sacroiliitis. In 2009, the Assessment of SpondyloArthritis international Society (ASAS) produced a classification of axSpA that includes patients with r-axSpA (also called AS) and nr-axSpA (Fig. 1) [3, 13–15]. The subsequent distinction between r- and nr-axSpA was driven by the historical concerns of regulators that nr-axSpA would be overdiagnosed and that spontaneous remission may be likely, making the benefit-risk ratio for treatment with tumor necrosis factor inhibitors (TNFis; which had been approved for AS) unfavorable [18]. Although nr-axSpA is currently classified as a separate condition, we anticipate that “axSpA” will become a universally accepted unitary term embracing both r- and nr-axSpA. This is not unlike how “seronegative” and “seropositive” or “erosive” and “nonerosive” rheumatoid arthritis are generally grouped together unless there is a specific reason to segregate them in a description of a patient or, for example, to suggest that a therapy may work better in a seropositive patient. Note that “nonradiographic” is a potentially confusing term since the patient with nr-axSpA may well have radiographic changes in axial and peripheral joints, including the sacroiliac joints—just not the radiographic changes needed to confirm AS. Additionally, since evidence of inflammation on magnetic resonance imaging (MRI) of the sacroiliac joints may be part of the definition of nr-axSpA, some clinicians may not focus on the fact that MRI is not a radiographic procedure, although it is an imaging procedure, leading to confusion about the terminology when MRI positivity is present.

**Fig. 2:**
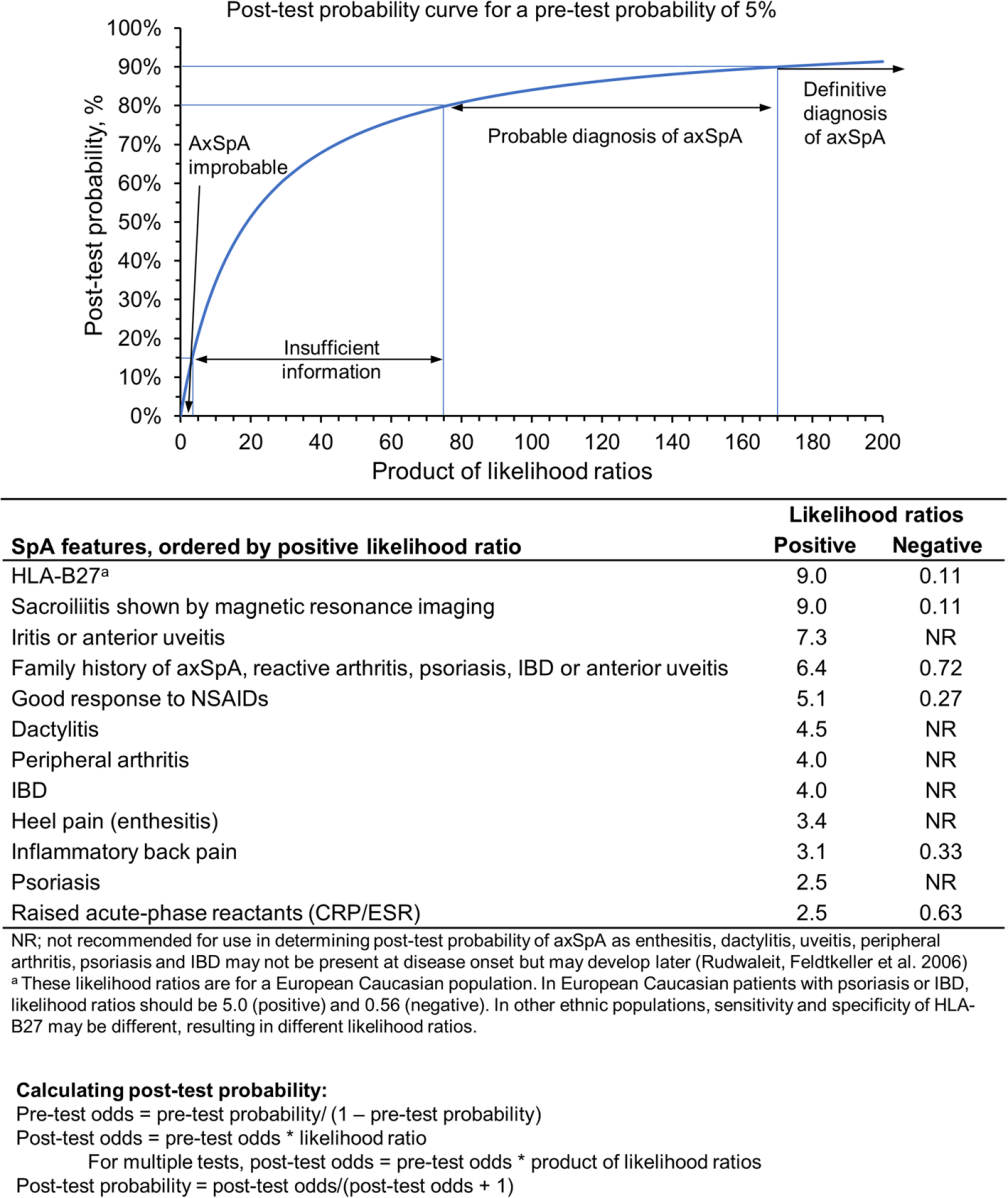
Likelihood ratios of SpA features in patients with axSpA*. axSpA = axial spondyloarthritis; CRP = C-reactive protein; ESR = erythrocyte sedimentation rate; HLA = human leukocyte antigen; IBD = inflammatory bowel disease; NSAID = nonsteroidal anti-inflammatory drug; SpA = spondyloarthritis. * Likelihood ratios include both positive and negative (where appropriate) likelihood ratios of SpA features in patients with axSpA and a method of determining the posttest probability of axSpA in patients with chronic back pain, assuming a prevalence of 5% in the group. Adapted from Rudwaleit M, Feldtkeller E, Sieper J. Easy assessment of axial spondyloarthritis (early ankylosing spondylitis) at the bedside. *Ann Rheum Dis*. 2006;65 (9):1251–1252. Copyright© 2006, BMJ Publishing Group Ltd. & European League Against Rheumatism [20, 21]

**Fig. 3:**
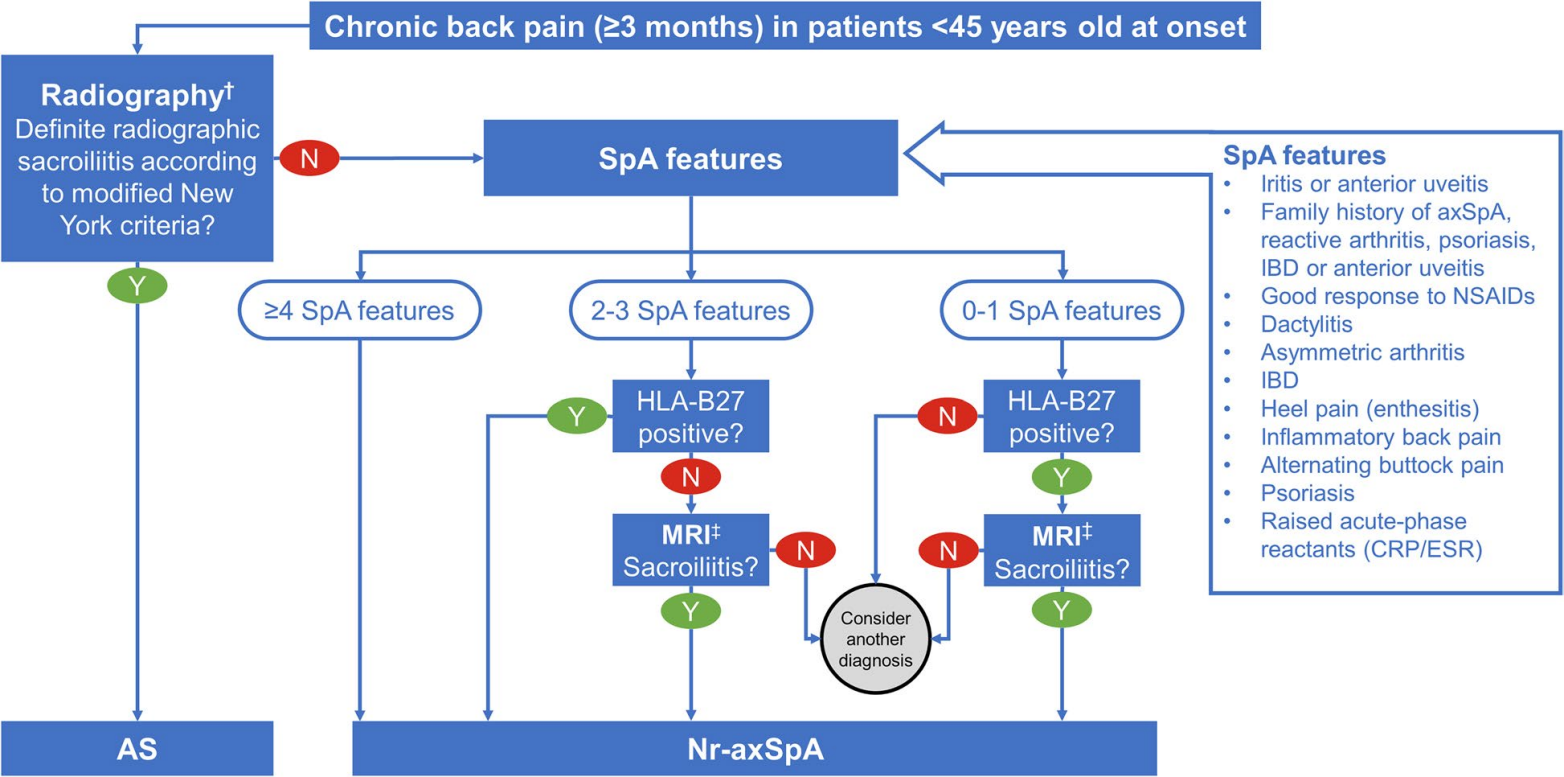
ASAS modification of the Berlin diagnostic algorithm for axSpA [22].* AS = ankylosing spondylitis; axSpA = axial spondyloarthritis; CRP = C-reactive protein; ESR = erythrocyte sedimentation rate; HLA = human leukocyte antigen; IBD = inflammatory bowel disease; MRI = magnetic resonance imaging; nr-axSpA = nonradiographic axial spondyloarthritis; NSAID = nonsteroidal anti-inflammatory drug; SpA = spondyloarthritis. * The algorithm is intended for use by rheumatologists for patients in the specified age range and not in an unselected population of patients with chronic back pain. ^†^ The modified New York criteria for sacroiliitis are: grade ≥ 2 bilaterally or grade ≥ 3–4 unilaterally [13]. ^‡^ Active acute inflammation is defined by clearly present bone marrow edema (on short-T1 inversion recovery) or osteitis (on T1 post gadolinium) highly suggestive of SpA, located in subchondral or periarticular bone marrow [15]

The classic presentation of nr-axSpA is onset of chronic lower back pain before 45 years of age [19]. However, we have found that younger patients with nr-axSpA may seek treatment for perceived sports injuries; peripheral skeletal pain such as entheseal pain around the knees, ankles, or feet; or similar types of manifestations—not reporting backache until a careful history is taken. Along with backache, patients may present with a wide variety of symptoms related to conditions associated with SpA (uveitis, dactylitis, peripheral arthritis, IBD, enthesitis, and psoriasis; see SpA features in Figs. 1-3) [20–22].

### Assessment and diagnosis of nr-axSpA

Diagnosis of nr-axSpA is a clinical judgement based on the pattern recognition, clinical reasoning, and summation of evidence by an expert rheumatologist [18, 23, 24]. In taking the patient’s history, attention should be paid to signs and symptoms of inflammatory back pain (eg, improvement with exercise, pain/waking up at night, alternating buttock pain, insidious onset, age of onset < 40 years, and no improvement with rest) [16, 17], the response to nonsteroidal anti-inflammatory drugs (NSAIDs), and prior symptoms of other SpA-related conditions. A family history of SpA-related conditions (axSpA, psoriasis, reactive arthritis, uveitis, or IBD) in first- and second-degree relatives should also be noted. Physical examinations for sacroiliitis are not practical, but patients should be examined for other SpA-related features (eg, uveitis, dactylitis, peripheral arthritis, enthesitis, and psoriasis).

Assessing human leukocyte antigen B27 (HLA-B27) status is important as positivity is strongly associated with axSpA [25]. Globally, the prevalence of HLA-B27 varies along racial lines (from 0.5% in Japanese to up to 25 to 50% in Inuit, Yupik, and Haida populations [26]) and generally mirrors the local incidence of SpA-related diseases [27]. In 3 recent international clinical trials in patients with nr-axSpA, between 63.6 and 83.5% of included patients were HLA-B27 positive [28–30]. In the United States, the prevalence (95% CI) of HLA-B27 is 6.1% (4.6–8.2%) in the general population, 7.5% (5.3–10.4%) in non-Hispanic White individuals, and 3.5% (2.5–4.8%) in all other races/ethnicities combined [31].

Laboratory tests for inflammatory markers (C-reactive protein and erythrocyte sedimentation rate) may be helpful, although their sensitivity (50%) for axSpA is modest [20].

The sacroiliac joint should be imaged by radiography; if nr-axSpA is suspected in the absence of radiographic sacroiliitis, MRI should be performed. MRIs need to be assessed carefully as a substantial proportion of healthy individuals (23%), without current or past back pain, have an MRI positive for sacroiliitis [32] using the 2016 ASAS MRI Working Group criteria [33]. The recently updated 2019 ASAS MRI Working Group consensus definitions for MRI lesions in the sacroiliac joint of patients with SpA require clearly present bone marrow edema (on short-T1 inversion recovery) or osteitis (on T1 post gadolinium) highly suggestive of SpA, located in subchondral or periarticular bone marrow [15]. Bone marrow edema that extends > 1 cm from the subchondral bone, is present in > 1 location, and is evident on ≥2 consecutive slices is highly suggestive of axSpA [33]. Presence of bone marrow edema alone does not meet the criteria for a positive MRI; however, findings should be interpreted in the context of presence of other structural lesions (eg, fat metaplasia and erosions) to increase specificity for a diagnosis of axSpA [33]. Deep and extensive bone marrow edema lesions are almost exclusively found in patients with axSpA [32]. More recently, a data-driven approach to defining a positive MRI has been proposed, using cut-offs for definite active and structural lesions that are highly predictive of a long-term clinical diagnosis of axSpA that can be used in a clinical setting [34].

Motivated by long delays in the diagnosis of AS, rheumatologists developed strategies for diagnosing early AS (ie, when sacroiliitis was not radiographically evident), which may be of utility in the diagnosis of nr-axSpA. The original Berlin diagnostic algorithm gave rise to a system combining the positive/negative likelihoods of individual signs and symptoms to calculate a posttest probability of axSpA (Fig. 2) [20, 21] and to the ASAS modification of the Berlin diagnostic algorithm (sensitivity, 78.5%; specificity, 79.6%; Fig. 3) [22], which were both intended for use by rheumatologists.

For simplified screening decisions in nonrheumatology practices, we recommend that patients < 45 years old with chronic back pain (≥3 months) are referred to a rheumatologist for suspected nr-axSpA if they have ≥1 of: HLA-B27 positivity, current inflammatory back pain, or sacroiliitis on MRI (sacroiliitis on plain radiography would indicate suspicion of AS) [19]. This simple strategy for referral has been shown to be effective and noninferior to more complex strategies in screening patients for axSpA, including those with nr-axSpA (44–55% of referred patients were diagnosed with definitive or possible axSpA) [35, 36].

### Differentiating nr-axSpA from its mimics

The diagnosis of nr-axSpA is complicated by the lack of definitive diagnostic criteria, the absence of specific blood biomarkers, and the wide variety of conditions that can lead to chronic backache. Differential diagnosis of nr-axSpA and appropriate referrals to rheumatologists are essential if the long delays in diagnosis are to be reduced.

Some conditions mimic nr-axSpA and several, notably fibromyalgia (also known as nociplastic pain or central sensitization), can co-occur. In our experience, the most common mimics of nr-axSpA and confounders of diagnosis are fibromyalgia and mechanical back pain. The most important mimics of nr-axSpA and their differentiating features are listed in Table 1 [37–54] and described below.

**Table 1:** Mimics that may confound the diagnosis of nr-axSpA

Condition	Possible confounding signs and symptoms	Differentiating features
Fibromyalgia (central sensitization) [37–42]	• Chronic back pain • Tenderness mimicking enthesitis • Possibly co-occurring	• Back pain generally gets better with rest and worse with activity • No relief with NSAIDs • No objective inflammatory disease on MRI • No objective inflammatory disease in musculoskeletal system or in the eyes, gut, or skin • Very high patient-reported disease activity after treatment for nr-axSpA
Degenerative disc disease [43–45]	• Possibly co-occurring, including in younger adults • Asymptomatic degenerative changes on MRI may be blamed for nr-axSpA symptoms (nr-axSpA mimicking disc disease)	• Not improved by exercise • Improved rather than worsened by rest • Radicular pain below the knee • Disc space narrowing on plain radiographs • Spinal bone marrow edema and fatty lesions across the whole endplate rather than just the anterior or posterior corners of the vertebrae on MRI • Changes concentrated in the lumbar spine rather than distributed across the whole spine • No sacroiliitis on MRI
Spinal osteoarthritis [46]	• Stiffness • Possibly co-occurring in older patients	• Not improved by exercise • Improved rather than worsened by rest • No sacroiliitis on MRI
Fractures		
Spondylolysis/spondylolisthesis [47]	• Chronic back pain	• Back pain generally gets better with rest and worse with activity • Pars defect or shifted vertebral body on lateral radiograph • Back pain greater in hyperextension • No sacroiliitis on MRI
Sacral fracture	• Chronic back pain	• Fracture on radiograph or MRI
Less common mimics		
Septic sacroiliitis [48–50]	• Subacute onset of back pain • Unilateral sacroiliitis	• Periarticular muscle edema as strongest predictor of differential diagnosis • Thick capsulitis (> 5 mm) • Extracapsular fluid collection • Large bone erosion
Scheuermann kyphosis [51]	• Chronic back pain with onset in early adolescence	• Back pain generally gets better with rest and worse with activity • Vertebral anterior wedge deformity on lateral radiograph • Schmorl nodes (disc herniation through the vertebral endplate) • No sacroiliitis on MRI
Spinal astrocytoma [52]	• Chronic back pain with insidious onset	• Back pain generally gets better with rest and worse with activity • Asymmetrical spinal cord expansion on MRI • No sacroiliitis on MRI
Familial Mediterranean fever [53, 54]	• Chronic back pain • Sacroiliitis • Peripheral arthritis • Enthesitis	• History of intermittent fever

*MRI* magnetic resonance imaging, *nr-axSpA* nonradiographic axial spondyloarthritis, *NSAID* nonsteroidal anti-inflammatory drug

#### Fibromyalgia

Fibromyalgia is an idiopathic syndrome characterized by widespread musculoskeletal pain that is thought to be a clinical manifestation of central sensitization [37, 40]. Fibromyalgia can both mimic the symptoms of nr-axSpA (back pain, tenderness mimicking enthesitis) as a differential diagnosis and be a comorbidity in patients with axSpA; presence of fibromyalgia does not exclude the diagnosis of nr-axSpA [37, 42].

The criteria for classification and diagnosis of fibromyalgia have evolved over time, resulting in substantial differences in the estimates of prevalence (0.4 to > 11%) and the proportion of female patients (≤60 to > 90%) [38, 55]. There is recent evidence that clinical diagnosis of fibromyalgia has been susceptible to bias, leading to underdiagnosis in men and overdiagnosis in women [56, 57].

A higher prevalence of fibromyalgia (10 to 30%) has been reported in patients with rheumatologic diseases [37], and a recent meta-analysis reported a 16.4% (95% CI, 12.3–20.5%) prevalence in patients with axSpA [39]. The latter study also reported a 20.3% (95% CI, 6.5–34.1%) prevalence of fibromyalgia in patients with MRI-positive nr-axSpA and an 11.1% (95% CI, 6.0–16.2%) prevalence in patients who met the ASAS clinical criteria for nr-axSpA but did not have positive MRIs [39]. For this subgroup of patients with both nr-axSpA and fibromyalgia, management of disease that only targets inflammation may not be optimal, as the underlying fibromyalgia will continue to impact quality of life.

Isolated fibromyalgia can be differentiated from nr-axSpA by the lack of any objective evidence of inflammation in the musculoskeletal system (eg, synovitis, enthesitis) or elsewhere (eg, uveitis, psoriasis, IBD) and an absence of inflammatory sacroiliitis on MRI [41, 42]. Concomitant fibromyalgia is important to diagnose since it has a strong effect on patient-reported outcome measures [40]. For example, very high Bath Ankylosing Spondylitis Disease Activity Index scores (≥8/10 in 3 of the first 5 questions) should increase suspicion of fibromyalgia [40]. Validated, multidimensional measures of central sensitization, including the Widespread Pain Index and Symptom Severity Scale, can be used to evaluate whether patients likely have fibromyalgia and are being adopted in real-world settings, such as the CorEvitas SpA/PsA Registry, to better characterize patients for future analyses [58].

#### Mechanical back pain

The term “mechanical back pain” is used to describe a symptom and is also used as a diagnosis. Unlike the symptoms of inflammatory back pain, the symptoms of mechanical back pain generally worsen with exercise and improve with rest [59]. It is important to remember that not all patients with nr-axSpA report back pain without being asked. A patient report of back pain that sounds mechanical in nature does not rule out nr-axSpA. Mechanical back pain is caused by structural changes that are initiated biomechanically, while nr-axSpA is caused by immune-mediated inflammation [59].

Degenerative disc disease, a common cause of mechanical back pain, may mimic nr-axSpA in the absence of associated radicular pain. Because degenerative changes on MRI have been found in a majority of patients with nr-axSpA across all ages [43], it is also important to recognize that nr-axSpA symptoms may be incorrectly attributed to asymptomatic disc degeneration [44].

Mechanical back pain due to spinal osteoarthritis is more likely in older patients; the prevalence of moderate or severe lumbar facet joint osteoarthritis is reported to be 36% in adults < 45 years old [46]. Co-occurring mechanical back pain may complicate the diagnosis of nr-axSpA in older adults.

#### Stress fractures

Stress fractures (or defects) of the pars interarticularis, causing spondylolysis and subsequent spondylolisthesis, are a potential source of chronic lower back pain [47]. Spondylolisthesis is easily identified on lateral radiographs, but spondylolysis may require computed tomography to confirm [47]. Pain on hyperextension has moderate sensitivity (81%) but low specificity (40%) in patients with spondylolysis [47].

Sacral stress fractures, which can occur post partum or as a sports injury in younger patients, can manifest as chronic lower back pain, which may present similarly to inflammatory back pain associated with sacroilitis [60, 61]. Sacral stress fractures can be discriminated by careful imaging studies and clarified through detailed patient history.

#### Less common mimics of nr-axSpA

Septic/infectious sacroiliitis is rare but can occur through multiple mechanisms (eg, brucellosis, tuberculosis, postpartum infection, posttrauma infection, fistula, and infection via injection sites) and with a variety of microorganisms (most commonly staphylococci) [48–50]. Septic sacroiliitis is generally unilateral and causes backache, the onset of which can be subacute. In addition, patients with septic sacroiliitis may have signs or symptoms of an infection, including fever, chills, or weight loss [49, 62]. It can be distinguished from SpA on MRI by the presence of thick capsulitis, extracapsular fluid collection, and periarticular muscle edema; periarticular muscle edema has been identified as the strongest predictor of a differential diagnosis of infectious sacroiliitis and SpA [48, 62].

Scheuermann kyphosis is a potential cause of chronic back pain with onset in early adolescence [51]. Lateral radiographs will show vertebral anterior wedge deformity, likely along with Schmorl nodes (disc herniation through the vertebral endplate) [51].

A spinal tumor or malignancy, the most common of which is spinal astrocytoma [52], may cause chronic lower back pain, and spinal astrocytoma can be distinguished by asymmetrical spinal cord expansion on MRI [52].

Patients with familial Mediterranean fever can exhibit several characteristics of nr-axSpA: back pain, sacroiliitis (more likely in HLA-B27–positive patients), peripheral arthritis, and enthesitis [53, 54]. These patients can by distinguished by a history of periodic fever [53, 54].

Clinicians should also be aware that other conditions affecting bone metabolism can mimic the radiographic signs of AS, although these conditions are not always considered in the differential diagnoses of early axSpA or nr-axSpA. Osteitis condensans ilii (OCI) is characterized by a triangular sclerosis on the iliac side of the lower end of the sacroiliac joints and can sometimes be confused for radiographic sacroiliitis [63]. MRI changes of osteitis in the sacroiliac joints may be similar between patients with OCI and axSpA; however, the prevalence of sacroiliac joint erosions is significantly higher in axSpA vs OCI, with erosions predominantly located in the middle of the sacroiliac joint for axSpA vs the anterior portion for OCI [64]. OCI is commonly seen in multiparous women, but it has been reported in nulliparous women and even in men [65].

Diffuse idiopathic skeletal hyperostosis (or Forestier disease) leads to the ossification of entheses and ligaments and mimics the syndesmophytes associated with AS, particularly in the spine [63]. Although the rate of new bone formation has been shown to be similar between diffuse idiopathic skeletal hyperostosis and AS, the absence vs presence of sacroiliitis has traditionally been considered a major distinguishing feature between the 2 diseases [63, 66, 67]. More recently, intra-articular joint ankylosis observed by computed tomography and enthesopathies in the axial skeleton have also been observed in patients with diffuse idiopathic skeletal hyperostosis [68, 69].

### Clinical practice pearls

The following are important considerations for clinical workup of patients with suspected nr-axSpA:

- Ask questions related to the clinical items defining inflammatory back pain: improvement with exercise, pain/waking up at night, alternating buttock pain, insidious onset, age of onset, improvement with NSAIDs, and no improvement with rest [16, 17]
  - Note that patients may assume that backache is part of the universal human condition and not mention it unless asked
  - Ask about the patient’s age at the onset of backache and about its persistency
- Obtain blood tests for inflammatory markers (C-reactive protein and erythrocyte sedimentation rate)
- Test for HLA-B27 (but be aware that not all patients with nr-axSpA, particularly those who are not White, are HLA-B27 positive)
- Increase suspicion of axSpA if conditions associated with SpA (uveitis, IBD, and psoriasis) are present along with backache
- Order imaging studies that include the sacroiliac joints—these are often missed as practitioners tend to focus on the lumbar spine
- Beware the potential pitfalls of a positive MRI—additional SpA features are required to confirm a diagnosis of nr-axSpA, although a newer data-driven approach to defining positive MRI has been proposed

### Management and treatment

The burden of disease experienced by patients with nr-axSpA is similar to that experienced by patients with AS, as is the response to treatment [70]. The most recent guidelines covering management and treatment of adults with nr-axSpA can be found in the 2019 update from the American College of Rheumatology (ACR), Spondylitis Association of America, and Spondyloarthritis Research and Treatment Network [71]. The ACR guidelines recommend physical therapy and first-line treatment with NSAIDs. For ongoing symptoms despite treatment with NSAIDs, the ACR guidelines recommend treatment with TNFis or interleukin 17A inhibitors (secukinumab or ixekizumab), with TNFis preferred as the first-line biologic.

Although certolizumab pegol and 3 other TNFis (adalimumab, etanercept, and golimumab) have been approved by the European Commission (EC) for the treatment of nr-axSpA, certolizumab was the first biologic approved for this use and is the only TNFi to be US Food and Drug Administration (FDA) approved for nr-axSpA after showing significant, sustained improvement over placebo in the signs and symptoms of nr-axSpA [30]. Clinical trials of interleukin 17A inhibitors, which were only recently FDA and EC approved for the treatment of nr-axSpA [72, 73], were ongoing at the time the most recent recommendations were written. The phase 3 COAST-X (ixekizumab) and PREVENT (secukinumab) studies recently reported 52-week results showing significant, sustained improvement over placebo in the signs and symptoms of nr-axSpA [28, 29].

For patients with suspected fibromyalgia, the patient’s response to treatment may further inform the diagnosis [40].

### Prognosis

Current treatments for nr-axSpA can reduce disease activity and improve patients’ physical function and quality of life [28–30]. Studies are now demonstrating the ability to achieve and sustain remission and are investigating maintenance of inactive disease following dose reduction or withdrawal and re-treatment upon disease flares [74–76].

## Conclusion

Nr-axSpA is not as uncommon as one might suppose from its relatively recent definition. Its prevalence appears to be approximately 0.5% in the general population, which means that it should be kept in mind by healthcare providers beyond the rheumatology specialty when assessing patients with chronic back pain and onset at < 45 years of age. Although the prevalence of nr-axSpA is similar between men and women, women present with more peripheral symptoms, are more likely to have widespread pain and/or fibromyalgia, and have fewer radiological abnormalities than men [77, 78]; as a result, nr-axSpA is often overlooked in women. Given the range and prevalence of mimics and the possibility of comorbid fibromyalgia, there is an art in sorting out overlapping symptoms and setting expectations for treatment.

Nr-axSpA is associated with a substantial burden of disease, and diagnostic delays can negatively affect patient outcomes, as untreated chronic inflammation may lead to irreversible damage. A variety of approved treatments are now available, and patients with nr-axSpA should be referred to rheumatologists for appropriate care.

## Data Availability

Data sharing is not applicable to this article as no datasets were generated or analysed during the current study.

## Abbreviations

ACR: American College of Rheumatology
ASAS: Assessment of SpondyloArthritis international Society
axSpA: Axial spondyloarthritis
HLA-B27: Human leukocyte antigen B27
IBD: Inflammatory bowel disease
MRI: Magnetic resonance imaging
nr-axSpA: Nonradiographic axSpA
NSAIDs: Nonsteroidal anti-inflammatory drugs
OCI: Osteitis condensans ilii
r-axSpA: Radiographic axSpA
SpA: Spondyloarthritis
TNFi: Tumor necrosis factor inhibitor
